# Chronic alcohol consumption-induced alterations in gut microbiota disrupt the integrity of the intestinal barrier

**DOI:** 10.3389/fmicb.2026.1851136

**Published:** 2026-07-28

**Authors:** Zizheng Gao, Weiyao Sun, Zilong Lun, Xiaoting Chen, Wei Wang, Yan Zhang, Zhuang Li, Chun Yang

**Affiliations:** 1 College of Basic Medicine, Beihua University, Jilin, China; 2 School of Public Health, Beihua University, Jilin, China; 3 Microbiome Medicine Center, Department of Laboratory Medicine, Zhujiang Hospital, Southern Medical University, Guangzhou, China

**Keywords:** Ccr2, gene expression, chronic alcohol consumption, gut microbiota dysbiosis, Intestinal barrier dysfunction, isoflavone metabolism

## Abstract

Chronic alcohol consumption is known to compromise the intestinal barrier and disrupt gut homeostasis, contributing to increased vulnerability to various diseases; however, the precise mechanisms remain poorly understood. Emerging evidence suggests that alcohol-induced alterations in host metabolism and gut microbiota composition play a critical role in driving intestinal barrier dysfunction. In this study, we employed a mouse model of chronic alcohol gavage to investigate these interactions. Untargeted metabolomics analysis revealed significant perturbations in isoflavone metabolism caused by chronic alcohol exposure. These metabolic disturbances were accompanied by notable shifts in gut microbiota composition, including an enrichment of *Colidextribacter* and *Ruminococcus*. Transcriptomic analysis further identified 286 upregulated and 367 downregulated genes in the alcohol group, and demonstrated that these microbial changes were strongly associated with the downregulation of the *Ccr2* gene in intestinal cells, implicating this pathway in intestinal barrier dysfunction. Taken together, these findings suggest that alcohol-induced disruptions in isoflavone metabolism contribute to gut microbiota imbalances, which were associated with suppressed *Ccr2* expression and impaired intestinal barrier integrity, although causal relationships and the specific cell types involved remain to be determined, providing new insights into the mechanisms underlying alcohol-related gut pathology.

## Introduction

Alcohol consumption is a major global health concern (Shield et al., 2020), with chronic intake significantly associated with severe physiological disturbances (Im et al., 2019, 2023), including intestinal barrier dysfunction and gut homeostasis imbalance. The intestinal barrier plays a fundamental role in maintaining host health by preventing the translocation of harmful microorganisms and toxins into systemic circulation while allowing the selective absorption of nutrients and metabolites (Blikslager et al., 2007; Groschwitz et al., 2009). Despite extensive research, the precise mechanisms by which alcohol compromises intestinal integrity and disrupts gut homeostasis remain poorly understood.

Emerging evidence highlights that alcohol-induced metabolic disturbances and alterations in gut microbiota composition are critical contributors to intestinal dysfunction (Gao et al., 2021; Zhao et al., 2023). Among these, disruptions in isoflavone metabolism have recently garnered significant attention in the context of alcohol-induced gut disturbances (Al-Nakkash et al., 2020). Isoflavones, dietary-derived secondary metabolites, are well known for their ability to regulate gut microbiota structure and modulate processes essential for host health (Ou et al., 2019; McCarty and Lerner, 2021). Studies suggest that alcohol severely interferes with isoflavone metabolic pathways, disrupting the balance between their key metabolites and the gut microbiota, thus exacerbating microbial dysbiosis. This metabolic imbalance has been linked to the enrichment of specific pathogenic bacteria and significant alterations in host gene expression, ultimately impairing the intestinal barrier. Nevertheless, the role of isoflavone metabolism in alcohol-induced gut pathology remains insufficiently explored.

Gut microbiota dysbiosis may play a pivotal role in alcohol-related intestinal dysfunction by modulating critical gut signaling pathways, such as the chemokine receptor *Ccr2*, which is essential for maintaining the integrity of the intestinal barrier (Emal et al., 2017; Liu et al., 2018; Koeninger et al., 2020; Mok et al., 2024). In this study, we utilized a chronic alcohol gavage mouse model to investigate the interplay between isoflavone metabolism and gut microbiota regulation, focusing on the molecular mechanisms underlying alcohol-induced intestinal barrier dysfunction. Through untargeted metabolomics, 16S rRNA sequencing, and transcriptomic analysis, we revealed that alcohol-induced disruptions in isoflavone metabolism are strongly associated with alterations in gut microbial composition, particularly with the enrichment of *Colidextribacter* and *Ruminococcus*. These microbial changes correlated with the downregulation of intestinal *Ccr2* expression. These findings provide novel insights into how isoflavone metabolism mediates alcohol-related gut dysfunction by influencing microbiota composition and gene regulation, advancing our understanding of alcohol-induced intestinal pathology.

## Materials and methods

### Materials and reagents

Alcohol was purchased from Guangzhou Chemical Reagent Factory (Guangzhou, China). The enzyme-linked immunosorbent assay (ELISA) kit for lipopolysaccharide (LPS) was obtained from ELK Biotechnology (Wuhan, China), and the ELISA kit for D-lactic acid (D-LA) was acquired from MEIMIAN (Jiangsu, China). Antibodies against Claudin-4, Occludin, and ZO1 tight junction proteins were procured from Abcam (Shanghai, China). All other chemicals and reagents were of analytical grade.

### Animals and animal experiment

The mice used in this experiment were 8 week-old wild-type C57BL/6J male mice, purchased from Zhuhai BesTest Bio-Tech (Zhuhai, China) and bred in the SPF-level experimental animal house at Zhujiang Hospital, Southern Medical University (Guangzhou, China). The breeding environment was maintained at 25 ± 1 °C with a regular 12 h/12 h light/dark cycle, high-pressure water, and SPF-grade mouse feed. All animal experiments were approved by the Ethics Review Committee of the Experimental Animal Center at Zhujiang Hospital, Southern Medical University (LAEC-2024-039), and conducted in strict accordance with the guidelines for the care and use of laboratory animals.

**Figure 1:**
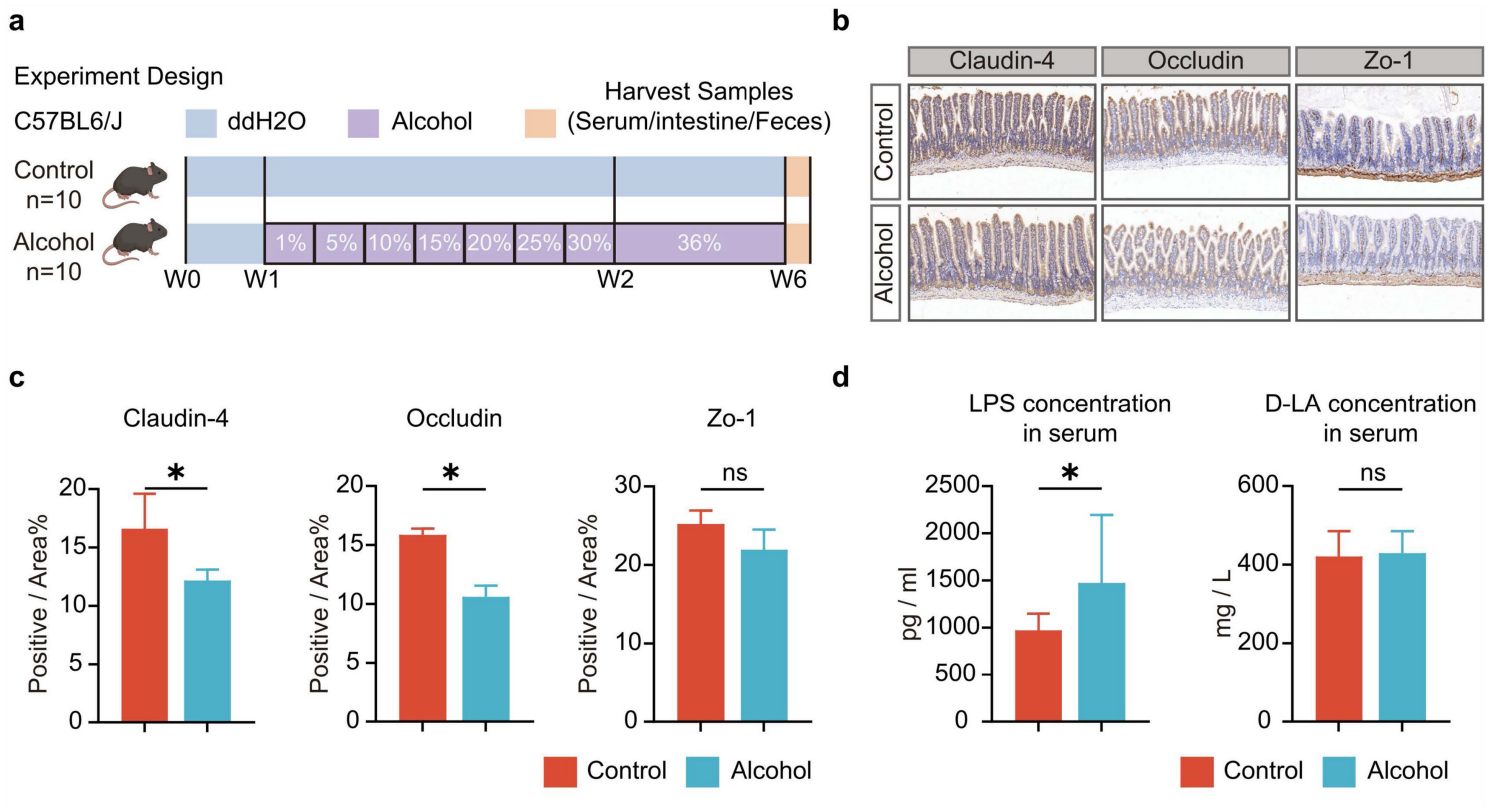
Chronic alcohol consumption compromises intestinal barrier integrity. **(a)** The schematic of animal experiment; **(b)** Representative images of Claudin-4, Occludin, and ZO-1 IHC staining are shown on sections of the proximal jejunum from control and alcohol-treated groups; **(c)** The quantification of Claudin-4, Occludin, and ZO-1 positive areas in mouse jejunal sections from control (*n* = 10,*P < 0.05) and alcohol-treated (*n* = 10,*P < 0.05) groups; **(d)** The concentration of LPS and D-LA in the serum are analyzed by ELISA in control (*n* = 10) and alcohol-treated (*n* = 10,*P < 0.05) mouse.

To investigate the impact of chronic alcohol consumption on intestinal integrity, mice in the alcohol group were first acclimated to the new environment with sterile water by daily gavage for 1 week. Subsequently, they received a daily gavage of alcohol solution at a starting concentration of 1% (v/v), which was increased by 5% (v/v) each day until reaching 36% on day 8. This 36% concentration was then maintained for an additional 4 weeks, resulting in a total treatment period of 6 weeks. The control group received sterile water daily throughout the entire period. Each gavage volume was 0.2 mL, prepared by diluting alcohol with sterile water. Body weight loss data were not collected in the current study, but this parameter will be monitored and included in future investigations (Figure 1a). Following the final gavage and completion of modeling, all mice were fasted for 12 h, with both food and water withheld. Cecum contents and ocular blood were then collected for subsequent assays, and small intestine tissues were rapidly excised for additional experiments.

A total of 20 male C57BL/6J mice were used in this study and randomly assigned to two groups (alcohol group and control group, *n* = 10 per group). All 10 mice per group were used for immunohistochemistry (IHC), serum ELISA (LPS and D-LA), untargeted metabolomics, 16S rRNA gene sequencing, and transcriptomic analysis. The sample size (*n* = 10 per group) was determined based on previous studies investigating intestinal barrier function and gut microbiota alterations in chronic alcohol mouse models, which typically used 8-12 mice per group to achieve sufficient statistical power for detecting significant differences in key outcome measures. No a priori power calculation was performed.

### Immunohistology

Under deep anesthesia, mice were euthanized, and segments of the jejunum were rapidly excised and rinsed with saline. The tissue was then fully embedded in paraffin and sectioned into 5-μm slices using a rotary microtome (Leica). The sections underwent antigen retrieval with citric acid (pH 6.0, G1202, Servicebio) and endogenous peroxidase was blocked with 3% hydrogen peroxide solution (D40036, Annjet). After blocking with 3% BSA in 0.1 M PBS, the sections were incubated overnight at 4°C with primary antibodies against claudin-4 (1:200, ab15104, Abcam), Occludin (1:200, ab216327, Abcam), and ZO-1 (1:500, ab221547, Abcam). Following washes, the sections were incubated with HRP-conjugated secondary antibodies (1:200, GB23303, Servicebio) for 50 min at room temperature. Color development was achieved using DAB, and hematoxylin was used for nuclear staining. Finally, the sections were dehydrated, mounted, and imaged using a microscope (E100, Nikon). The slides were scanned using the Pannoramic MIDI II digital section scanner (3DHISTECH), and the percentage of positive cells was calculated using ImageJ software.

### Measurement of serum inflammatory factor

Following sacrifice, blood was collected from the mouse and centrifuged at 3,500 rpm for 10 min at 4 °C. The serum was then isolated for detecting inflammatory cytokines. LPS and D-LA levels were determined using ELISA kits in accordance with the manufacturer's instructions.

### Untargeted metabolomics

The freshly collected intestinal contents from each treatment group were immediately snap-frozen in liquid nitrogen and subsequently stored at −80 °C. The samples were submitted to Majorbio Bio-Pharm Technology Co., Ltd. (Shanghai, China) for untargeted metabolomics analysis. For each sample, 3 μL of solution was separated at 40 °C using a Waters ACQUITY HSS T3 C18 column (100 mm × 2.1 mm I.D., 1.8 μm) on a Vanquish Ultra-Performance Liquid Chromatograph (Thermo Fisher Scientific). The mobile phase, consisting of a 0.1% formic acid aqueous solution (phase A) and acetonitrile (phase B), was pumped at a flow rate of 0.4 mL/min. The following gradient was employed: a linear increase from 0 to 20% B (0-4.5 min), 20 to 35% B (4.5-5 min), 35 to 100% B (5-6.3 min), followed by isocratic elution at 100% A for 0.1 min, and finally a return to 0% B for 1.6 min.

Full scan and fragmentation analyses were performed using a high-resolution Exploris 240 Orbitrap mass spectrometer (Thermo Fisher Scientific). Global operating parameters were as follows: a spray voltage of 3,500 V in HESI positive ion mode and −3500 V in HESI negative ion mode; a transfer tube temperature of 325 °C; and a source temperature of 425 °C. The sheath gas and auxiliary gas flow rates were set to 50 Arb and 13 Arb, respectively. Data-dependent acquisition (DDA) was conducted with a survey scan range of 70-1050 m/z, an MS1 resolution of 60,000, and subsequent MS/MS scans at a resolution of 7,500. High-purity nitrogen was used as both the atomization gas and the collision gas for high-energy collisional dissociation.

The raw data were first converted to mzXML format using MSConvert in the ProteoWizard software package (v3.0.8789) and processed with XCMS for feature detection, retention time correction, and alignment. Multivariate data analysis and modeling were performed using Ropls software. After data scaling, models were built using orthogonal partial least-square discriminant analysis (OPLS-DA). To correct for systematic bias, robust LOESS signal correction (QC-RLSC) was applied for data normalization. Post-normalization, only ion peaks with relative standard deviations (RSDs) below 30% in quality control samples were retained to ensure accurate metabolite identification. Differential metabolites were subjected to pathway analysis with MetaboAnalyst, which integrates pathway enrichment analysis with pathway topology analysis. Identified metabolites were then mapped to the KEGG pathway for biological interpretation of systemic functions. Finally, metabolites with a *P* value < 0.05 and VIP values >1 were considered statistically significant.

### Determination of microbiota in Cecal contents

Since the cecal bacterial content was abundant, fecal samples could be obtained. The freshly collected intestinal contents from each treatment group were immediately snap-frozen in liquid nitrogen and stored at −80 °C. The samples were submitted to Majorbio Bio-Pharm Technology Co., Ltd. (Shanghai, China) for high-throughput sequencing of the 16S rRNA V3-V4 hypervariable region. Total bacterial genomic DNA was extracted from the fecal samples using the FastPure Stool DNA Isolation Kit (MJYH, Shanghai, China) according to the manufacturer's instructions. The extracted DNA was assessed by 1% agarose gel electrophoresis and a NanoDrop NC2000 ultraviolet spectrophotometer for concentration and quality. A 5 μL sample was used to determine the DNA concentration and OD260/280 ratio. The V3-V4 region of the extracted DNA was amplified by PCR using the forward primer 338F (5′-ACTCCTACGGGAGGCAGCAG-3′) and the reverse primer 806R (5′-GGACTACHVGGGTWTCTAAT-3′). The 20 μL PCR reaction mixture comprised 4 μL of 5 × reaction buffer, 2 μL dNTP (2.5 mM), 0.8 μL forward primer (5 μM), 0.8 μL reverse primer (5 μM), 2 μL DNA template, 10 μL ddH_2_O, and 0.4 μL TransStart FastPfu DNA polymerase. The target fragments were separated by 2% agarose gel electrophoresis. The recovered products were quantified using the Synergy HTX (BioTek, USA) and sequenced on the NextSeq 2,000 PE300 platform. The function of the 16S rRNA was predicted using PICRUSt2 (version 2.2.0), and gut microbiota was analyzed by 16S rDNA amplicon sequencing on the Majorbio Cloud platform (https://cloud.majorbio.com) in both control and alcohol-treated mice.We have deposited the 16S rRNA sequencing data in the NCBI database (PRJNA1479103).

### Transcriptome sequencing experiment and analysis

Freshly collected jejunal tissue samples from each treatment group were immediately snap-frozen in liquid nitrogen and subsequently stored at −80 °C. The samples were then submitted to Beijing Novo gene Co., Ltd. for transcriptome sequencing and analysis. RNA quantification of jejunal tissue samples was conducted using a Bioanalyzer 2100 system (Agilent Technologies, CA, USA). The RNA was then reverse transcribed to synthesize cDNA for library construction, and the library fragments were purified using the AMPure XP system (Beckman Coulter, Beverly, USA). Index-coded sample clustering was performed on a cBot Cluster Generation System with the TruSeq PE Cluster Kit v3-cBot-HS (Illumina) according to the manufacturer's instructions. Following cluster generation, the library preparations were sequenced on an Illumina NovaSeq platform, yielding sequencing data for all samples with a read length of 150 bp (paired-end).

Prior to analysis, raw data underwent quality control. FeatureCounts v1.5.0-p3 was employed to count the number of reads mapped to each gene. Subsequently, the FPKM (fragments per kilobase of exon per million mapped reads) of each gene was calculated based on its length and the number of reads mapped to it. Differential expression analysis between two groups was performed using DESeq2. Gene Ontology (GO) enrichment analysis of differentially expressed genes was implemented by the clusterProfiler R package, in which gene length bias was corrected. Similarly, Kyoto Encyclopedia of Genes and Genomes (KEGG) pathway enrichment analysis was performed using the clusterProfiler R package to test the statistical enrichment of differential expression genes.

### Data statistics

Statistical analyses were performed using Prism 9.5 (GraphPad Software Inc., USA). Data are presented as the mean ± standard error of the mean (SEM). Differences between groups were assessed using Student's *t*-test, with *P* < 0.05 considered statistically significant.

## Results

### Chronic alcohol consumption compromises intestinal barrier integrity

To investigate the impact of chronic alcohol consumption on intestinal integrity, mice were gavaged daily with 36% ethanol for 6 weeks, while controls received sterile water (Figure 1a). Immunohistochemical staining revealed a significant reduction in jejunal tight junction proteins Claudin-4 and Occludin, with Zo-1 showing a non-significant downward trend (Figures 1b, c). Additionally, serum analysis showed a significant increase in LPS levels, a biomarker of gut permeability, in alcohol-treated mice, while D-lactic acid levels remained unchanged (Figure 1d). These findings demonstrate that chronic alcohol exposure compromises intestinal barrier function by reducing tight junction protein expression and increasing gut permeability.

### Chronic alcohol consumption alters gut metabolism and enhances isoflavonoid biosynthesis

**Figure 2:**
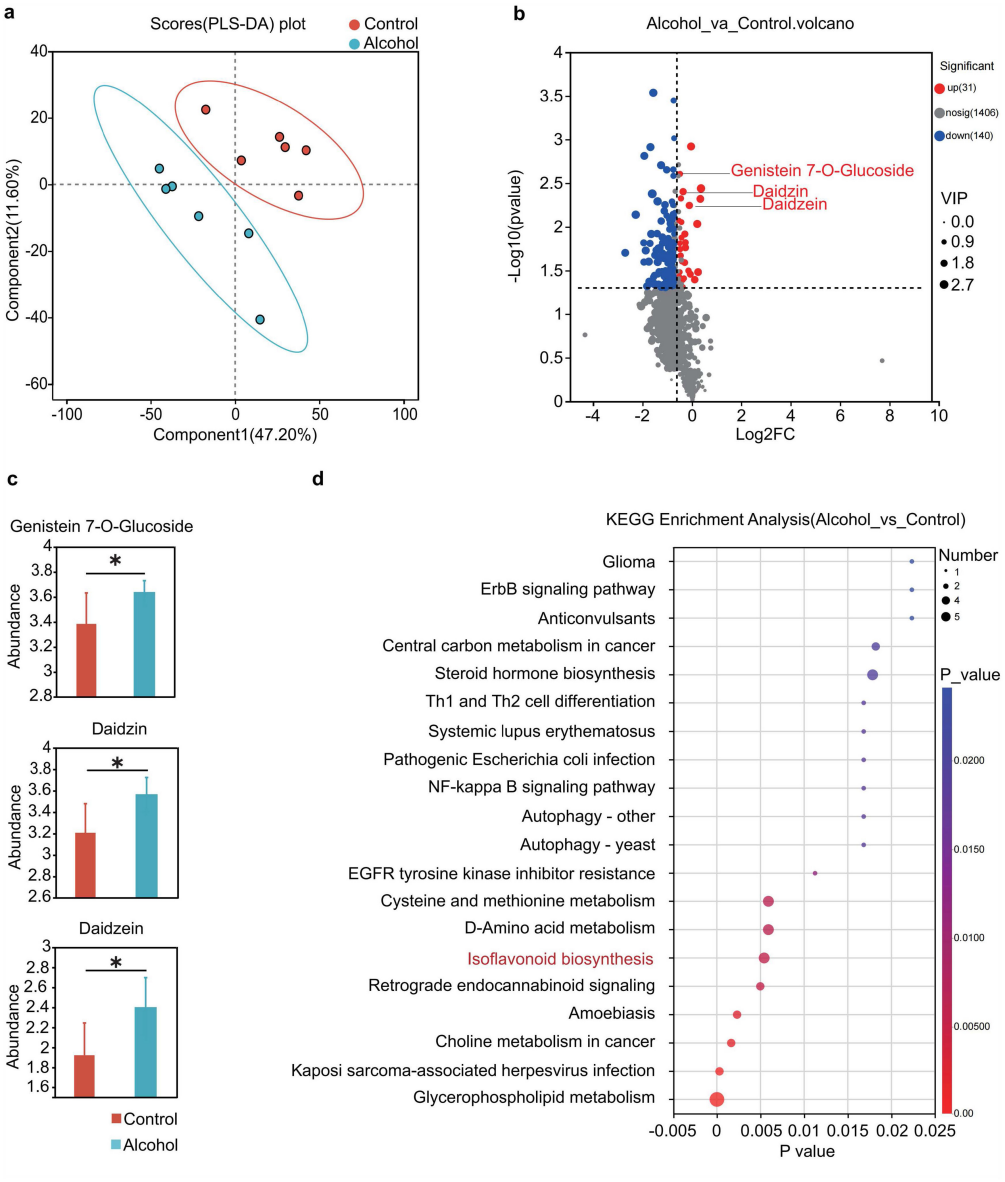
Chronic alcohol consumption alters gut metabolism and enhances isoflavonoid biosynthesis. **(a)** Untargeted metabolomic analysis demonstrates the distinction in chronic alcohol metabolites; **(b)** The alterations in metabolites following chronic alcohol were analyzed utilizing volcano plot visualization; **(c)** Targeted bar plot analysis revealed chronic alcohol perturbations in [daidzin, genistein 7-O-glucoside, and daidzein] abundance (e.g., *n* = 10, *P < 0.05,); **(d)** Untargeted metabolomic of KEGG analysis with top20 significance.

To examine the impact of chronic alcohol consumption on host gut metabolism in mice, a non-targeted metabolomics analysis of cecal contents was conducted. Orthogonal partial least squares-discriminant analysis (OPLS-DA) revealed clear clustering and significant separation between the Alcohol and Control groups along the Component 1 and Component 2 axes, suggesting distinct metabolic profiles between the two groups (Figure 2a). Volcano plot analysis, using variable importance in projection (VIP) > 1 and *p* < 0.05 as filtering criteria, identified 31 metabolites upregulated and 140 metabolites downregulated in the Alcohol group compared to the Control group (Figure 2b). Notably, the metabolites daidzin, genistein 7-O-glucoside, and daidzein were significantly upregulated in the Alcohol group (Figure 2c). To explore these metabolic changes further, all identified differential metabolites were subjected to Kyoto Encyclopedia of Genes and Genomes (KEGG) enrichment analysis, which highlighted significant alterations in the Isoflavonoid biosynthesis pathway (*p* < 0.01) between the Alcohol and Control groups (Figure 2d). These results demonstrate that chronic alcohol consumption leads to a pronounced increase in the levels of daidzin, genistein 7-O-glucoside, and daidzein, accompanied by enhanced activity in isoflavone biosynthesis.

### Chronic alcohol consumption induces gut microbiota shifts correlated with altered metabolite profiles

**Figure 3:**
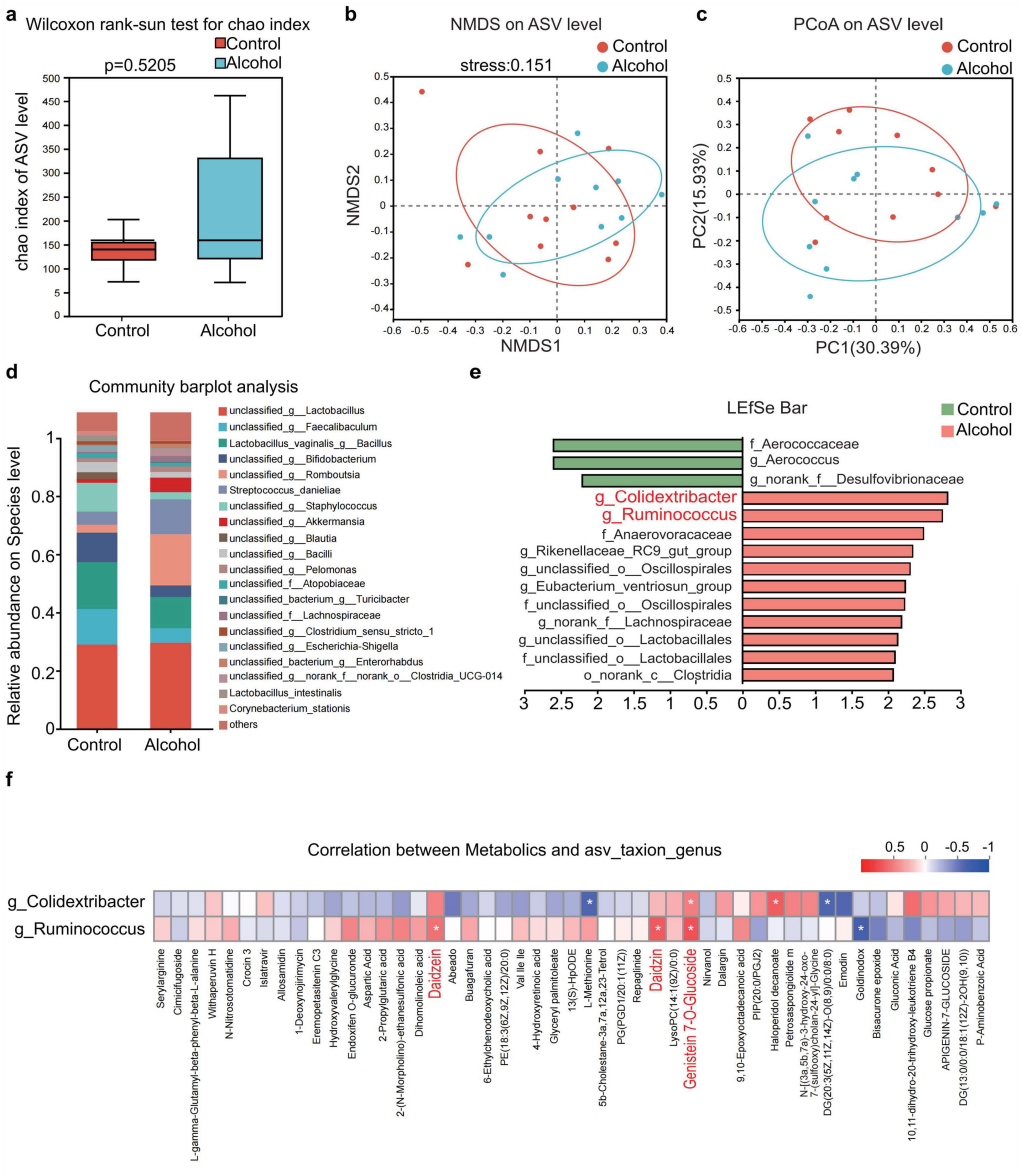
Chronic alcohol consumption induces gut microbiota shifts correlated with altered metabolite profiles. **(a-c)** Chronic alcohol intake triggered dysbiosis of the gut microbiota, as evidenced by 16S rRNA-based α & beta-diversity analysis; **(d)** Comparative analysis of microbial community structure at the genus level revealed distinct taxonomic profiles between the Alcohol-exposed and Control cohorts (*n* = 10); **(e)** LEfSe analysis (LDA score >2.5) identified *Colidextribacter* and *Ruminococcus* as alcohol-associated biomarker genera; **(f)** Joint analysis reveals the correlation between metabolites and *Colidextribacter* and *Ruminococcus* (*P < 0.05).

To examine the effects of chronic alcohol consumption on gut microbiota composition in mice, 16S rRNA sequencing was conducted. The Chao1 index, a measure of α-diversity, showed no significant differences among the groups (Figure 3a). However, principal coordinates analysis (PCoA) and non-metric multidimensional scaling (NMDS) based on Bray-Curtis distances revealed distinct clustering of gut microbiota between groups (Figures 3b,c). At the genus level, significant differences in microbial composition were observed, with *Aerococcus* enriched in the Control group, while *Colidextribacter* and *Ruminococcus* were significantly more abundant in the Alcohol group (Figures 3d,e). Joint analysis of differential metabolites and bacterial abundance revealed positive correlations between the levels of daidzin, genistein 7-O-glucoside, and daidzein, and the bacterial genera *Colidextribacter* and *Ruminococcus* in the Alcohol group (Figure 3f). These findings suggest that chronic alcohol consumption leads to shifts in gut microbiota composition, potentially driven by altered metabolite profiles.

### Chronic alcohol consumption alters gut microbiota and downregulates *Ccr2*, compromising gut barrier integrity

**Figure 4:**
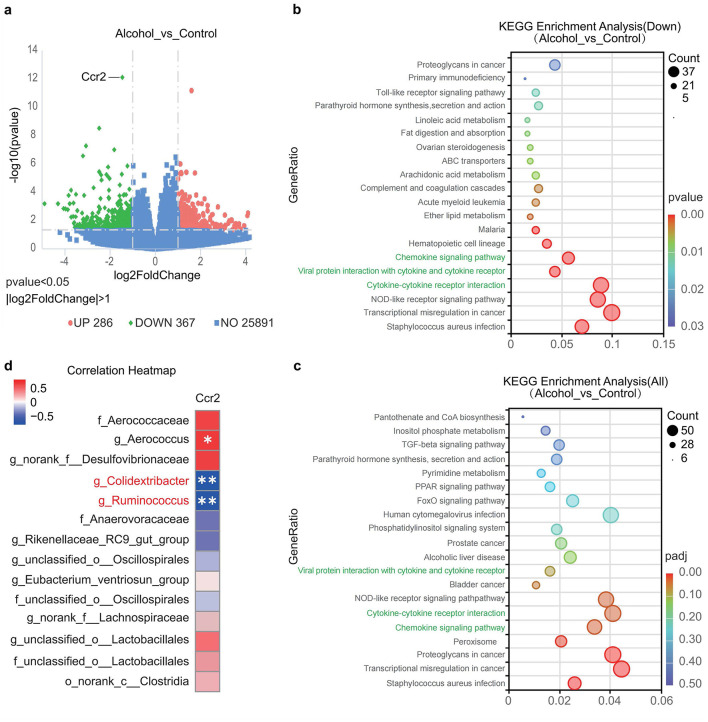
Chronic alcohol consumption alters gut microbiota and downregulates *Ccr2*, compromising gut barrier integrity. **(a)** The alterations in genes following chronic alcohol were analyzed utilizing volcano plot visualization; **(b, c)** Transcriptomic sequencing of KEGG analysis with top20 significance (upregulated and downregulated); **(d)** Joint analysis reveals the correlation between Gut microbiota and *Ccr2*(*P < 0.05, **P < 0.01).

To determine whether alterations in gut microbiota resulting from chronic alcohol consumption induce genetic changes, transcriptomic analysis was performed. Volcano plot analysis identified 286 upregulated and 367 downregulated genes in the Alcohol group, with the *Ccr2* gene being the most significantly downregulated (Figure 4a). KEGG enrichment analysis revealed significant downregulation of pathways including “Viral protein interactions with cytokines and cytokine receptors,” “Cytokine-cytokine receptor interaction,” and “Chemokine signaling,” with the *Ccr2* gene serving as a key component in these pathways (Figures 4b, c). Furthermore, correlation analysis demonstrated a negative association between the increased abundance of *Colidextribacter* and *Ruminococcus* and the downregulation of the *Ccr2* gene (Figure 4d). These findings suggest that the enrichment of *Colidextribacter* and *Ruminococcus*, driven by chronic alcohol consumption, is a critical factor contributing to the downregulation of the *Ccr2* gene, ultimately compromising immune defenses and impairing gut barrier integrity.

## Discussion

This study is exploratory and hypothesis-generating in nature, aiming to identify potential associations rather than establish causation. Our findings demonstrate correlational associations between chronic alcohol consumption and gut barrier dysfunction. Specifically, chronic alcohol exposure was associated with increased isoflavonoid metabolites (daidzin, genistein 7-O-glucoside, and daidzein), enrichment of *Colidextribacter* and *Ruminococcus*, and suppression of *Ccr2* gene expression. These changes were associated with weakened immune defenses and increased intestinal permeability. These associations suggest a potential link, but causal relationships remain to be established through future intervention studies.

Isoflavonoids exhibit a dual effect on intestinal homeostasis. While moderate levels of isoflavonoids can improve intestinal barrier function, excessive accumulation has detrimental effects, such as dysregulating cytokines, including increased IL-17A expression. Elevated IL-17A levels intensify inflammatory responses and disrupt intestinal stability (Kojima et al., 2015; Wu et al., 2020). Chronic alcohol consumption may impair isoflavonoid metabolism by depleting ethanol dehydrogenase (ADH) and acetaldehyde dehydrogenase (ALDH) activity, potentially leading to excessive accumulation of isoflavonoids and further exacerbating gut microbial dysbiosis. Of note, control mice (Figure 2c) showed detectable but significantly lower levels of these metabolites, confirming that the standard SPF diet (typically containing soy-based protein) is a source of isoflavones. The marked elevation in alcohol-treated mice therefore suggests alcohol-induced metabolic dysregulation rather than increased dietary intake alone. However, we acknowledge that direct quantification of dietary isoflavone content is needed to definitively establish this point. Additionally, the activation of the microsomal ethanol oxidation system (MEOS), which relies on cytochrome P450 enzymes, disrupts isoflavonoid metabolic balance (Chen et al., 2013). Daidzin, an ALDH2 inhibitor, worsens alcohol-induced reductions in tight junction (TJ) and adherens junction (AJ) proteins, increasing permeability and further undermining gut barrier integrity (Rungratanawanich et al., 2023).

Alterations in the gut microbiota further amplify intestinal barrier disruption. In particular, *Ruminococcus*, a pro-inflammatory microbe enriched following alcohol consumption, has been shown to impair barrier function by increasing intestinal permeability. This genus produces inflammatory polysaccharides that stimulate dendritic cells to secrete tumor necrosis factor (TNF-α), exacerbating inflammation (Aldred et al., 1999). Chronic alcohol exposure heightens the sensitivity of Kupffer cells to TNF-α, compounding *Ruminococcus*-induced gut inflammation and damage. Conversely, reducing *Ruminococcus* abundance has been associated with improved intestinal barrier integrity (Kim et al., 2017; Peng et al., 2022; Silverman et al., 2022).

Similarly, *Colidextribacter*, a lipopolysaccharide (LPS)-producing gut microbe significantly enriched in the alcohol group, disrupts tight junctions and contributes to increased intestinal permeability and barrier dysfunction. Elevated *Colidextribacter* abundance has been linked to impaired tight junction protein expression, further compromising intestinal barrier integrity (Zhu et al., 2023; He et al., 2024; Li et al., 2024). Together, our correlational data suggest that *Ruminococcus* and *Colidextribacter* may act as key drivers of alcohol-induced gut dysfunction, potentially through their pro-inflammatory and barrier-compromising effects. However, direct mechanistic studies (e.g., gnotobiotic colonization or *in vitro* models) are required to establish a causal role for these bacteria.

At the molecular level, chronic alcohol consumption caused marked suppression of *Ccr2* gene expression. The *Ccr2* gene is critical for recruiting monocytes to inflammation sites, reinforcing immune defenses, and maintaining intestinal integrity (Mok et al., 2024). Its downregulation impaired key cytokine and chemokine signaling pathways, further weakening barrier maintenance mechanisms. Reduced *Ccr2* expression also increased intestinal permeability, enabling LPS translocation into the systemic circulation, which exacerbates inflammation and systemic damage (Sun et al., 2001; Ceni et al., 2014; Koeninger et al., 2020; Di Vincenzo et al., 2024). These results align with previous studies showing that chronic alcohol consumption adversely impacts *Ccr2* expression and gut immune function (Hoebinger et al., 2023; Samuelson et al., 2023; Villageliu et al., 2024).

Regarding the discrepancy between D-lactate (D-LA) and lipopolysaccharide (LPS) as permeability markers in this study, we acknowledge that D-LA is a well-established biomarker of intestinal barrier dysfunction in alcohol models. However, we did not observe a significant increase in serum D-LA levels after chronic alcohol exposure, whereas LPS levels were significantly elevated. This discrepancy may be explained by differences in the origin, kinetics, and clearance mechanisms of these two markers. D-LA is primarily produced by gut bacteria and rapidly cleared by the host; its levels may peak at earlier time points or in response to acute rather than chronic alcohol insults. In contrast, LPS is a structural component of Gram-negative bacterial cell walls, and its sustained translocation into the circulation during long-term alcohol exposure may better reflect persistent barrier impairment. Notably, the significant elevation of LPS, together with the reduced expression of tight junction proteins Claudin-4 and Occludin, consistently supports the conclusion that chronic alcohol consumption compromises intestinal barrier integrity in our model. Although D-LA did not show a significant change, the collective evidence from LPS, tight junction proteins, and gut microbiota shifts provides a coherent picture of alcohol-induced gut barrier dysfunction. Future studies incorporating multiple time points and additional permeability markers will help further elucidate these dynamic changes.

A major limitation of this study is that the central mechanistic claim—that alcohol-induced isoflavonoid accumulation promotes bacterial enrichment, which in turn suppresses *Ccr2* expression—is derived entirely from correlational analyses. No intervention experiments were performed to establish causation. Specifically, we did not conduct (a) isoflavone depletion experiments to determine whether removing dietary isoflavones abrogates the observed effects; (b) bacterial supplementation or depletion experiments (e.g., gavage with *Colidextribacter* or *Ruminococcus*, or antibiotic depletion) to test whether these bacteria directly drive barrier dysfunction; or (c) *Ccr2* knockout or overexpression models to validate the functional role of *Ccr2* in alcohol-induced gut injury. Therefore, our findings should be interpreted as hypothesis-generating rather than conclusive. Future studies incorporating these intervention approaches are necessary to establish causality.

Several additional limitations should be acknowledged. First, this study used only male mice. Sex-specific differences in alcohol metabolism, immune responses, and gut microbiota composition are well documented. Therefore, our findings may not be directly generalizable to females, and future studies should include both sexes to investigate potential sex-dependent effects of chronic alcohol exposure on the isoflavone-microbiota-*Ccr2* axis. Second, our metabolomics and 16S rRNA sequencing were performed on cecal contents, while transcriptomic analysis was conducted on jejunal tissue. Although cecal microbial metabolites can influence distal jejunal gene expression via systemic circulation, this regional mismatch represents a biological limitation. Third, our transcriptomic analysis was performed on bulk whole jejunal tissue, which contains multiple cell types. Therefore, the downregulation of *Ccr2* cannot be unequivocally attributed to intestinal epithelial cells or barrier function specifically. Fourth, although *Ccr2* was identified as the most significantly downregulated gene by transcriptomic analysis, we did not validate these changes at the protein level using techniques such as Western blot, immunofluorescence, or flow cytometry. Therefore, we cannot confirm whether the observed mRNA downregulation translates into reduced *CCR2* protein expression. Future studies employing region-matched sampling, gnotobiotic models, cell type-specific transcriptomics (e.g., single-cell RNA sequencing), and protein-level validation will be necessary to validate and extend our findings.

In conclusion, chronicalcohol consumption is associated with disruptions in intestinal barrier homeostasis, correlated with excessive metabolite accumulation, microbiota dysbiosis, and impaired gene regulation. The findings of this study highlight potential isoflavonoid-mediated microbial and genetic pathways in alcohol-induced gut dysfunction. However, given the correlational nature of our data, these findings should be interpreted as hypothesis-generating rather than conclusive. Future research should focus on therapeutic interventions to restore isoflavonoid metabolic balance, moderate microbiota composition, and enhance *Ccr2* expression to mitigate intestinal barrier damage and related health implications. Causal validation will require intervention studies including isoflavone depletion, bacterial supplementation or depletion, and *Ccr2* knockout/overexpression models.

## Conclusions

In this study, our results suggest that chronic alcohol exposure in mice is associated with perturbations in isoflavone pathways, enrichment of specific bacteria such as *Colidextribacter* and *Ruminococcus*, and downregulation of the *Ccr2* gene in the gut. These findings identify an alcohol-gut axis, linking dietary metabolite metabolism to microbial dysbiosis and impaired barrier integrity. This reveals potential therapeutic targets, such as the *CCR2* pathway or isoflavone supplementation, for treating alcohol-related gut diseases and preventing systemic complications in patients.

## Data Availability

The data presented in the study are deposited in the BioProject repository, https://www.ncbi.nlm.nih.gov/sra/PRJNA1479103.
